# The Role of Quantification of Glucocorticoid-associated Toxicity in Severe Asthma

**DOI:** 10.33696/immunology.3.074

**Published:** 2021

**Authors:** PJ McDowell, JH Stone, LG Heaney

**Affiliations:** 1 Centre for Experimental Medicine, School of Medicine, Dentistry, and Biological Sciences; Queen's University Belfast, Northern Ireland, UK; 2 Division of Rheumatology, Allergy, & Clinical Immunology, Massachusetts General Hospital,Harvard Medical School, USA

**Keywords:** Severe asthma, Glucocorticoid toxicity index (GTI)

## Introduction

Until recently, oral glucocorticoid (GC) therapies were the mainstay of treatment for uncontrolled inflammatory disease across many body systems. The last 30 years, however, have witnessed a transformation in the management of many diseases due to the development of targeted biological agents leading to a reduction, albeit not a removal, of the dependence on oral GCs. The need for new glucocorticoid-sparing therapies was urgent given the ever increasing and robust literature on the multisystem adverse effects related to chronic or repeated oral GC exposures [1–6]. The importance of measuring the potential GC-toxicity reduction by “steroid-sparing” agents — and the absence of a validated tool with which to accomplish this goal — was recognised by a group of international investigators from a broad range of medical specialities. These investigators developed and validated such a tool, the Glucocorticoid Toxicity Index (GTI) [7].

The purpose of the GTI is to quantify toxicity change over time following the introduction of a steroid-sparing agent. A systematic assessment of the full range of toxicities is undertaken on starting a steroid-sparing agent, and is repeated following a designated treatment interval, e.g., six or twelve months. The severity of each domain of toxicity is scored according to pre-determined, weighted scores for every toxicity item [7,8]. The GTI was designed for and is employed in randomised controlled trials, but because toxicity reduction is the key aim of using steroid-sparing agents in routine clinical care, systematic documentation of changes in GC toxicity at the level of individual patients in practice is also advantageous.

## The Use of Biological Agents in Severe Asthma

The Global Initiative of Asthma (GINA) reports the incidence of asthma is between 1–18% of the population in different countries [9], affecting an estimated 334 million people worldwide [10], with around 4% having severe asthma that remains uncontrolled despite treatment optimisation [11]. Eosinophilic asthma is driven by T2 cytokines (predominantly IL5, IL4 and IL13) that cause differentiation of eosinophils in the bone marrow, migration into the bloodstream, chemotaxis into the airways, and activation with release of inflammatory mediators, resulting in airways inflammation and injury [12,13]. This T2-driven inflammation can result in chronically uncontrolled asthma symptoms, but can also cause acute asthma flares or “exacerbations”. Eosinophilic inflammation is suppressed by GC therapy, and for the majority of patients this is via inhaled corticosteroids (ICS), but daily oral glucocorticoids (‘maintenance oral GC’) were previously required for those with ongoing symptoms from airways inflammation despite ICS optimisation, and short courses of oral GC for the treatment of acute asthma exacerbations (GC ‘boosts’) [9]. The addition of oral GC was the mainstay of treatment for ongoing airways inflammation in severe asthma resulting in approximately 60% of those in specialist severe asthma services in the UK receiving maintenance oral GC [14].

Approval of mepolizumab for the treatment of severe, eosinophilic asthma by the U.S. Food & Drug Administration (FDA) in 2015 marked a global turning point in the treatment of severe asthma. Biological agents are designed to interrupt biological pathways known to cause disease. In this case, mepolizumab binds to IL5, inhibiting maturation, activation and longevity of eosinophils, thereby reducing airway inflammation and diminishing reliance upon oral GCs. There are now two agents which bind IL5 (mepolizumab [15,16], reslizumab [17]), an anti-IL5 receptor antagonist (benralizumab [18]), and more recently an anti-IL4 a receptor antagonist which blocks IL4 and IL13 signalling (dupilumab [19]) approved for the treatment of asthma. These monoclonal antibodies target specific parts of the T2 cytokine pathway and have demonstrated an impressive 36–54% [15–20] reduction in oral GC-requiring exacerbations in placebo-controlled trials. These agents have also facilitated significant reduction in maintenance GC requirements in those requiring maintenance GC for asthma control prior to biologic therapy [21–23].

The question of how to measure a significant clinical response to these expensive therapeutic agents for severe asthma is actively being considered. Currently in the UK, the National Institute for Health Care Excellence (NICE) has suggested clinical review up to one year following the initiation of a biological agent. This guideline permits continuation of the biologic if there has been a “clinically significant reduction in GC-requiring exacerbations” or a “clinically-significant reduction in continuous oral GC” (50% reduction in the case of mepolizumab). However, no consensus exists with regard to the definition of “clinically significant reduction” — either in the number of exacerbations or in cumulative oral GC dose — and there is no mention of reducing GC toxicity.

The multisystem adverse effects associated with GC use in severe asthma are well documented, and constitute not only a significant economic burden to healthcare systems, but also an enormous personal burden to patients [2,3,5,24–26]. To date, these GC toxicities have been described only at a population level. Such data, although informative, have limited utility in assessing toxicity burden at the level of the individual patient. Moreover, they do little to ensure the active management of GC toxicities and the mitigation of risk from these toxicities in routine care. The aim of novel steroid-sparing agents is to achieve asthma control while minimising reliance on GC, thereby minimizing treatment-associated morbidity. Yet systematic quantification of GC toxicity has been absent from clinical trials assessing the effectiveness of new steroid-sparing agents. To understand toxicity change following the start of such an agent, an accurate measurement of GC toxicity burden at an individual patient level prior to treatment with a steroid-sparing agent is essential.

## GTI Assessment of Glucocorticoid Associated Toxicity

To explore GC toxicity at the level of individual patients with severe asthma, we used the validated Glucocorticoid Toxicity Index (GTI) [7] to assess toxicity systematically in a consecutive cohort of 101 severe asthma patients^8^. All patients met criteria for eligibility for mepolizumab therapy at a severe asthma specialist clinic in the UK. Our aims were:

1. to quantify the burden of GC toxicity in individual patients;
2. to assess the spread of GC toxicity across the population;
3. to elicit the factors that influence GC toxicity most strongly;
4. to report toxicities not previously identified;
5. to determine the minimal clinically important difference of toxicity change in GTI scores; and,
6. to describe the development of the web-based GTI app (GTI 2.0).

The cohort had substantial symptom and quality-of-life burdens from their asthma. The mean Asthma Control Questionnaire-5 (ACQ-5) score was 2.6 (1.3); the mean mini-Asthma quality-of-life questionnaire (mini-AQLQ) score was 3.6 (1.4); and the mean Saint George’s respiratory questionnaire score (SGRQ) was 55.8 (20.9). All of these measures confirm the severity of patients’ baseline asthma. Not surprisingly, the cohort had significant GC exposure in the 12 months preceding the initial GTI assessment: 82.2% had received maintenance oral prednisolone (median 10mg/day [10,15]) and the median annual number of prednisolone boosts for asthma exacerbations was 5 [interquartile range 2, 7]. In sum, median prednisolone exposure was 11.7 mg/day [interquartile range 8.4, 15.0] and annual cumulative prednisone exposure was 4280 mg/year [interquartile range 3082.5, 5475.0].

Given the scale of GC exposure, a high prevalence of GC toxicity in these patients at baseline was expected. The most common manifestations of GC-toxicity were reflected in neuropsychiatric disturbances (81.2%), skin toxicities (79.2%), and elevated body mass indices (69.3%). Metabolic disturbances were also frequent: impaired glucose metabolism (HbA1C ≥ 5.7%) was present in 65.3% (≥ 6.0% in 43.5%) and 67.3% were hypertensive. All patients had some degree of GC toxicity with GTI scores ranging from 33–377 (mean 177.5 (73.7) [8], but the distribution of toxicity observed at the individual patient level was wide.

Patient access guidelines define the need for steroid-sparing agents on the basis of recent GC exposure. However, in this cohort of biologic-naïve patients who met stringent national prescribing criteria in the UK, we show that GC-toxicity described by the GTI score correlated only modestly with recent prednisolone exposure (cumulative prednisolone exposure over the preceding 12 months and GTI score, Spearman’s correlation rho=0.38, p=<0.001). When performing multiple linear regression using variables thought to be clinically relevant to GTI toxicity (namely age, gender, cumulative GC exposure in the last 12 months and patient reported outcomes (mini-AQLQ), only age and mini-AQLQ were significant contributors to GTI toxicity. Each increasing year of age was associated with an increase in GTI score, likely to in part reflect the importance of the duration of GC exposure and possibly an increase in background incidence of many GC-toxicities with age.

Understanding the minimal clinically important difference (MCID) is important to the application of any clinical instrument. The MCID for the GTI was calculated using data from patients studied in the original GTI development [8], employing a distribution-based approach to derive the MCID [27]. Definition of this MCID will be important for interpreting the GTI data which is currently in use in 30 clinical and trial settings. The online GTI 2.0 application (GTI 2.0 app© 2016, 2018. Massachusetts General Hospital. All rights reserved) was developed to facilitate the scoring of the GTI. Patients’ data are entered quickly into the web-based application, thus facilitating data collection, maintaining a longitudinal record of GC toxicities, and ensuring rigor in GTI scoring. The GTI 2.0 app reduces user error and enables the handling of vast amounts of anonymised data. (For further information on the GTI application see https://www.crestlabsgti.com/. A worked example of the GTI app can be viewed at https://www.youtube.com/watch?v=Pz4CSwZe7eI.

## Conclusion

In summary, assessment of GC-toxicity requires assessment both in the clinical setting as well as in trials designed to assess GC reduction with steroid-sparing therapies. Measuring GC-toxicity in a systematic manner in individuals aids optimisation of clinical care and management of unrecognised toxicities. Further knowledge of what constitutes a significant toxicity change will help to inform the definition of a clinical response to a steroid-sparing agent in individual patients. Given that the purpose of these expensive biological agents is to minimise GC use, toxicity reduction should be part of the discussion around the definition of response. Moreover, toxicity change may be a more relevant determinant of clinical response than recent GC exposure. Finally, when baseline toxicity within a patient cohort is known, toxicity change may have a role in measuring efficacy of steroid-sparing agents at a population level, thus enabling head-to-head comparison between different agents.

The GTI is now in use across a number of diseases encompassing multiple sub-specialties. Important clinical trials using this instrument are now being completed [28–36]. Further prospective studies are required not only in clinical trials but also in routine care to ascertain how to optimise use of the GTI with the goal of reducing GC toxicity in our patients.
